# Well-being balance and lived experiences assessment: a valid, comprehensive measure of positive well-being

**DOI:** 10.3389/fpsyg.2024.1396543

**Published:** 2024-08-08

**Authors:** Ashley F. McDermott, Christopher R. Brydges, Troy W. Norris

**Affiliations:** ^1^WellBalance Institute, Boston, MA, United States; ^2^NIH West Coast Center, University of California, Davis, Davis, CA, United States

**Keywords:** subjective wellbeing, positive psychology, human flourishing, balanced well-being, wellbeing assessment, assessment validation, factor analysis

## Abstract

**Introduction:**

Widely used measures of self-reported subjective well-being and flourishing generally do not directly measure positive experiences that have been demonstrated to improve subjective well-being and flourishing, which could aid in developing personalized interventions to improve individuals’ well-being. The present study evaluated the validity of the Well-being Balance and Lived Experience (WBAL) Model and Assessment, a new model of well-being and corresponding assessment instrument that evaluates the self-reported frequency of positive experiences and positive feelings of well-being, balanced across activation and arousal levels.

**Methods:**

A total of 496 evaluable subjects completed the WBAL Assessment, the PERMA+ Profiler (PERMA+) and the Well-Being Assessment Adult 24-item (WBA-24). A confirmatory factor model corresponding to the WBAL construct was created, and internal and external validity of the WBAL Assessment were interrogated.

**Results:**

The confirmatory factor model showed good fit, indicating that each of the model factors are related but distinct and all items load significantly onto their factors. The WBAL Assessment demonstrated high internal consistency (Cronbach’s *α* = 0.95) and internal validity across well-being factors and Feelings (*r* = 0.96) and Experiences (*r* = 0.94) domains. The WBAL Assessment demonstrated strong convergent validity in comparison to PERMA+ (*r* = 0.80) and WBA-24 (*r* = 0.75), indicating that the WBAL Assessment measures a similar overall concept of well-being and flourishing. Discriminant validity of WBAL factors was demonstrated for an average of 14.3 of 17 comparator domains. The main differences between instruments are WBAL’s assessment of positive Experiences, the comparator instruments’ inclusion of feelings with negative valence, and WBA-24’s inclusion of financial stability.

**Discussion:**

The WBAL Assessment is a reliable and valid instrument to comprehensively measure positive aspects of well-being that evaluates multiple modifiable contributors to individuals’ well-being to guide design of personalized assessment and intervention programs to enhance positive well-being.

## 1 Introduction

Our understanding of well-being has advanced dramatically in recent years, in part due to the availability of measures that aim to capture the full complexity and multi-domainality of human well-being. Well-being extends upon the concept of “wellness,” which often connotes physical, mental, social and spiritual health, as a guide to counseling or medical intervention (Myers et al., 2000; Roscoe, 2009). Whereas wellness encompasses multiple health dimensions and lifestyle choices, well-being includes subjective evaluations of life satisfaction and personal fulfillment (Dodge et al., 2012).

There are a wide range of self-report instruments measuring well-being or closely related constructs (i.e., quality of life and wellness), which vary significantly in length, psychometric properties, and their conceptualization and operationalization of well-being (Kobau et al., 2010; Cooke et al., 2016; VanderWeele et al., 2020). Two of the primary conceptualizations of well-being are the hedonic and eudaimonic traditions. In the hedonic tradition, subjective well-being entails the balance of positive over negative feelings, alongside overall life satisfaction (Diener et al., 2009), encompassing the subjectivist focus on subjective life satisfaction. Measures of subjective well-being are based on the subjective experience of the individual, include positive measures of well-being, and assess all aspects of a person’s life (Diener, 1984).

In the eudaimonic tradition, which emphasizes positive functioning and achievement of a fulfilling and meaningful life, Seligman (2011) argues for greater focus on relationships, and accomplishment, and Jayawickreme et al. (2012) additionally propose including positive emotion, engagement and meaning (PERMA). Together, these domains are often measured alongside negative emotions, physical health, and loneliness (PERMA+).

VanderWeele (2017) proposes a summary measure of human flourishing that includes happiness and life satisfaction, mental and physical health, meaning and purpose, character and virtue, and close social relationships, with an additional domain of financial and material stability as a proxy for sustained flourishing (Flourish Index). Stiefel et al. (2020b) developed a similar summary instrument of well-being, which was subsequently integrated with Vanderweele’s flourishing domains to create a summary well-being assessment spanning life satisfaction and evaluation, physical and mental health, meaning and purpose, character and caring, relationships, community and social support, financial evaluation and stability, and overall affect (Well-Being Assessment).

These well-validated psychometric assessments of well-being have primarily been deployed as observational tools for evaluating the well-being of populations, whether for longitudinal epidemiological monitoring (Hone et al., 2014; Chen et al., 2022), comparisons across populations (Khaw and Kern, 2015; Weziak-Białowolska et al., 2019; Lee et al., 2022; Shiba et al., 2022), or evaluation of the impact of interventions on population well-being (Seligman et al., 2005; Ronzi et al., 2018; McTiernan et al., 2022).

Existing validated measures of well-being generally focus on subjective feelings without measuring lived experiences from which those feelings may arise, and over which individuals may have a degree of control to change their level of engagement. While these assessments have proven to be useful descriptive tools to assess well-being and monitor well-being longitudinally across populations, and can identify general domains of well-being around which to direct interventions, they have limited ability to support design of personalized interventions around specific positive experiences that have potential to improve well-being for individuals.

Psychological and physiological experiences of emotion are typically categorized using two categorical dimensions, emotional valence and arousal (Russell, 1980; Watson and Tellegen, 1985; Ekman, 1992; Barrett, 2006). Measures of subjective well-being and flourishing typically assess a range of emotional valence from positive to negative affect. Emotional arousal, a key dimension of core affect, describes the activation and energy toward some objective, usually in response to an experiential stimulus (Russell, 2003). Recent research has demonstrated the importance of lower arousal positive emotional states for well-being. For example, contentment has been shown to be a strong predictor of well-being and life satisfaction (Cordaro et al., 2024), and dispositional mindfulness and serenity are associated with lower stress and increased mental well-being (Soysa et al., 2021). While existing instruments typically contain multiple items related to emotions with moderate to high arousal levels, such as happiness or joy, they contain few items evaluating emotions with lower levels of arousal, such as contentment or satisfaction.

The Well-Being Balance and Lived Experiences (WBAL) Model of well-being, (summarized in Appendix A), builds upon and integrates the accumulated knowledge of hedonic and eudaimonic well-being to enable more comprehensive and granular evaluation of discrete, modifiable aspects of individuals’ positive well-being. By identifying specific categories of experiences more likely to improve feelings of well-being, the aim of the WBAL Assessment is to enable more efficient and effective development of personalized plans to improve positive well-being for individuals.

Specifically, the WBAL Model has been designed to extend the utility of previous well-being assessments along three dimensions:

1. Evaluate lived experiences that have been demonstrated to correspond with feelings of well-being;
2. Assess feelings and experiences with an even balance of low, moderate and high arousal and activation levels;
3. Include a full range of positive experiences and feelings previously demonstrated to contribute to positive well-being.

Respondents are prompted to subjectively self-assess the frequency of specific categories of positive experiences and positive feelings. Frequency is not an objective quantitative metric tailored to each prompt, but rather a subjective self-assessment on a 5-point Likert scale (rarely, sometimes, often, usually, very often). As a result, responses may be influenced by the respondent’s values and expectations of how frequently they should be engaging in a specific experience or feeling a certain emotion. And their recollection of the frequency of experiences and feelings may be influenced by their intensity (Cahill and McGaugh, 1995; Talmi et al., 2007) or subjective significance, representing the depth of an experience and importance in relation to the situation, goals and values of an individual (Imbir, 2016; Imbir et al., 2017). While this limits the ability to make quantitative comparisons across respondents, this approach more accurately reflects each respondent’s subjective evaluation of their experiences and feelings related to positive well-being.

The WBAL Model is thus a comprehensive integrative subjective construct of positive aspects of well-being that:

- expands the assessment of hedonic pleasures and positive affect by evaluating multiple distinct categories of positive feelings across the full range of emotional arousal levels, and
- extends the eudaimonic assessment of human flourishing by integrating positive experiences alongside subjective feelings associated with various aspects of fulfillment and satisfaction with life.

In order to embody these features within the limits of a feasible assessment tool, the WBAL Model focuses on the frequency of emotions with positive valence and does not directly evaluate emotions with negative valence, such as loneliness, anger or sadness. And the WBAL Model does not directly assess objective life situations, such as financial security, food security or physical disability, that may impact well-being or flourishing but are not specific feelings or experiences *per se*.

The objective of this study was to evaluate the internal reliability and validity of the WBAL Model and Assessment, and their external validity in comparison to PERMA+ and WBA-24.

## 2 Materials and methods

### 2.1 Materials

This study utilized three separate instruments to assess well-being, including the WBAL Assessment (WBAL), the PERMA+ Profiler (PERMA+), and the Well-Being Assessment for Adults 24-Item (WBA-24).

#### 2.1.1 WBAL assessment

The Well-Being Balance and Lived Experiences Assessment (WBAL Assessment or WBAL-30), shown in Table 1, measures the frequency of distinct items of positive Experiences and positive Feelings related to well-being in the WBAL Model. The WBAL Assessment has 30 items scored on a 5-point Likert Scale (from 0 to 4) measuring respondents’ self-reported subjective frequency of these positive Experiences and Feelings over the past 2 weeks (0 = Rarely, 1 = Sometimes, 2 = Often, 3 = Usually, 4 = Very Often).

**Table 1:** Well-being and lived experiences assessment instrument, 30-item (WBAL-30).

**Domain**	**Factor**	**Energy level**	**Item**	**#**	**Prompt**
**Experiences**		**Activation level:**			Over the past 2 weeks, how often have you had the following experiences? (0 = Rarely, 1 = Sometimes, 2 = Often, 3 = Usually, 4 = Very Often)
	**Body**	Active	Move Regularly	1	My days are physically active, I exercise regularly, and my body is strong and able.
		Mindful	Nourish Healthily	2	I savor nutritious food and eat only until full, while hydrating regularly without too much alcohol or caffeine.
		Calm	Rest and Recover	3	I sleep well and let myself rest and recover when I’m sore, injured or tired.
	**Mind**	Active	Create, Learn and Explore	4	I learn new things, express my creativity and become fully absorbed in activities.
		Mindful	Savor and Appreciate	5	I spend time in nature, and appreciate and enjoy music, art, and good stories.
		Calm	Reflect Gratefully	6	I pause to reflect, feel grateful and connect to something larger than myself.
	**Connection**	Active	Build Community	7	I engage with groups beyond my close friends and family, and seek out new people that share my interests.
		Mindful	Bond Closely	8	I regularly connect with my close friends or family and we help each other when needed.
		Calm	Love Securely	9	I spend undistracted time with a loving, trusted companion, and we listen to and meet each other’s needs.
	**Purpose**	Active	Contribute, Serve and Earn	10	I help make the world better, positively impact others, and am rewarded fairly for my work.
		Mindful	Provide and Nurture	11	I am responsible, provide for others’ wellbeing and help make my home comfortable and safe.
		Calm	Kindness and Grace	12	I am kind to others, supporting and comforting them, without judgment or resentment.
	**Activation Range**	Active	Active and Engaged	13	My body is active and fit, my mind is engaged, and I have a meaningful impact in my community
		Mindful	Mindful and Present	14	I pay attention to and take care of myself and others, am present in the moment and appreciate the world around me.
		Calm	Calm and Restful	15	My relationships are secure, I am physically safe, and I can relax and be at peace.
**Feelings**		**Arousal Level:**			Over the past 2 weeks, how often have you had the following feelings? (0 = Rarely, 1 = Sometimes, 2 = Often, 3 = Usually, 4 = Very Often)
	**Arousal Range**	Joyful	Joyful and Confident	16	My life feels meaningful and fun, filled with purpose, joy and laughter.
		Aware	Aware and Appreciative	17	I savor life’s special moments, am self-aware, and appreciate the people in my life.
		Content	Content and Peaceful	18	I feel content and satisfied with my life, at peace with myself and safe with others.
	**Openness**	Joyful	Adventurous and Curious	19	I enjoy meeting new people, exploring new cultures and trying new experiences.
		Aware	Harmonious and Attentive	20	I appreciate nature, art and music, and feel connected to people in my life and in harmony with my world.
		Content	Trusting and Safe	21	I trust myself and others to keep us safe, and believe things will work out.
	**Significance**	Joyful	Proud and Mattering	22	My life matters and has meaning, and I am proud of my accomplishments.
		Aware	Belonging and Accepted	23	I feel like I belong, am welcome and appreciated, and can be myself with people in my life.
		Content	Gentle and Loved	24	I feel loving kindness and am gentle towards others, and feel loved and cared for in return.
	**Efficacy**	Joyful	Capable and Confident	25	I feel confident and capable to contribute meaningfully and take care of myself and others.
		Aware	Considerate and Responsible	26	Others can depend on me and I feel able to provide for myself and others.
		Content	Caring and Compassionate	27	I care for and feel compassion towards myself and others.
	**Wellness**	Joyful	Vital and Strong	28	I feel alive and energetic, with a strong body and sharp mind.
		Aware	Satisfied and Fulfilled	29	I feel fulfilled and satisfied, appreciating small pleasures in the moment.
		Content	Peaceful and Serene	30	My life feels peaceful, serene and untroubled, with a restful body and calm mind.

Copyright WellBalance, LLC (2023); available for research use with permission.

WBAL scores represent averages of all items overall and within two domains of positive Experiences and positive Feelings. Twelve (12) categories of positive Experiences are mapped to four (4) factors of Mind, Body, Connection and Purpose, in addition to three (3) items evaluating the frequency and range of Experiences Activation levels. Twelve (12) categories of positive Feelings are mapped to four (4) factors of Wellness, Openness, Significance and Efficacy, in addition to three (3) items evaluating the frequency and range of Feelings Arousal levels. Each of the 10 total factors contains three (3) items representing low, moderate and high Arousal and Activation levels within the factor.

#### 2.1.2 PERMA+ profiler

The PERMA+ Profiler (Butler and Kern, 2016) was designed to measure the five pillars of well-being identified by Dr. Martin Seligman of the University of Pennsylvania (Hone et al., 2014). The five pillars are Positive emotion, Engagement, Relationships, Meaning, and Accomplishment. The full PERMA+ Profiler also measures happiness, health and vitality, negative emotion, and loneliness. The PERMA+ Profiler has 23 items scored on an 11-point Likert scale (from 0 to 10) that are mapped to 9 domains.

#### 2.1.3 Well-being assessment for adults 24-item

The Well-Being Assessment for Adults 24-item (WBA-24) was developed collaboratively by members of the Institute for Healthcare Improvement’s 100 Million Healthier Lives metrics team and The Human Flourishing Program at Harvard’s Institute for Quantitative Social Science (Stiefel et al., 2020a). This harmonized consolidation of well-being assessments incorporates both the Institute for Healthcare Improvement’s Well-Being Assessment for Adults 12-item (WBA-12; Stiefel et al., 2020b) and the Harvard Flourishing Index (Seligman, 2011), along with additional items developed jointly. Each item in these assessments is scored on an 11-point Likert scale (from 0 to 10), and the 24 items are mapped to 8 domains.

### 2.2 Participants

Study participants were recruited in the United States through SurveyMonkey Audience via Momentive.ai. Participants had to be between the ages of 20–69 and have a minimum income of $25,000 per year.

We planned to have 500 participants complete the full survey, with nested stratification across age groups and gender, with a target of 100 respondents (census-balanced for gender, i.e., 48 male, 52 female) in each age group of 20–29, 30–39. 40–49, 50–59, 60–69 years. A power analysis indicated that at alpha = 0.05, having 500 evaluable responses would be able to detect correlations greater than 0.125 with 80% power (*p*-value <0.01) and greater than 0.145 with 90% power (p-value <0.001).

Payment was set and provided by Momentive and was set to be a nominal amount less than one US dollar ($1) per respondent.

### 2.3 Procedure

Study participants were recruited in the United States by Momentive.ai from SurveyMonkey Audience panels, which are proprietary and exclusive, are composed of a diverse group of people generally reflective of the US population, regularly calibrated to ensure high response quality, and members of whom have opted in to participate in research projects. The study survey instrument was 80 questions long which respondents completed in approximately 9 min. It included the questions from the Well-Being Balance and Lived Experiences Assessment (WBAL-30), the PERMA+ Profiler (PERMA+), and the Harvard/IHI Well-Being Assessment (Adult-24 Item; WBA-24) in a set order.

The study was determined by Solutions IRB to present no or minimal risk to research subjects and therefore exempt from ongoing IRB oversight. A statement of voluntary consent appeared at the beginning of the survey emphasizing that the subject’s participation is completely voluntary, they are not expected to benefit from participating, and they can stop participating at any time during the survey.

### 2.4 Evaluable subjects

A total of 496 participants were included in the analyses as evaluable subjects. Momentive automatically screened out potential bots or fraudulent responses based on email and location verification. Momentive also used ID exclusions to prevent duplicate responses. After this initial screening, there were 646 total respondents. Two responses were incomplete and excluded from analysis. An additional 115 responses were identified as “speeders” based on completion times being less than 2/3 of the median completion time for the survey (i.e., <5.9 min vs. 8.8 min median time to completion). An additional 33 responses were removed from the analysis as “cheaters” whose responses on the comparator survey questions were all within a tight range (+/− 1 point on 0–10 scale) and had answers to the reverse coded items in the same range, indicating that the respondents were not reading and accurately responding to specific questions.

Of the 496 evaluable subjects, 284 (57%) were female and 212 (43%) were male, 92 (19%) were ages 18–29, 151 (30%) were 30–44, 158 (32%) were 45–60, and 95 (19%) were 61–69. The income distribution was reflective of the US income distribution with 389 (78%) participants making less than $100,000 per year.

### 2.5 Analysis

To confirm the overall validity of the WBAL Model, a confirmatory factor analysis (CFA) was conducted in IBM SPSS Amos 27 (Arbuckle, 2020). Specifically, three alternative models of increasing complexity were compared to determine the best fit for the data. The first model was a one-factor model, where all 30 items loaded onto a single wellness factor. Secondarily, a correlated 10-factor model was evaluated for which three items representing each energy level within each factor were loaded onto the four Feelings factors, the four Experiences factors, the Feeling Arousal energy range factor, and the Experience Activation energy range factor. These 10 factors were free to correlate with each other.

The third and final model was the same as the correlated 10-factor model, but with the residual variances of each energy level free to correlate with each other. For example, the residual variances of the high energy items (active/engaged and joyful/confident) were free to correlate with each other, but not with items from the mid (mindful/present and aware/appreciate) and low (calm/restful and content/peaceful) energy items. Similarly, the residual variances of the mid energy items were free to correlate amongst themselves, but not with the high or low energy items, and the low energy items were free to correlate amongst themselves, but not with the high or mid energy items. This model was posited to account for the commonalities in energy levels across factors.

Prior to any CFA analysis, 19 participants were identified as multivariate outliers when examining Mahalanobis distances (critical value of *p* < 0.001) and were excluded from all models. Based on guidelines provided by Schweizer (2010), the CFA models were considered to have acceptable model fit if they met all the following criteria: Comparative Fit Index (CFI) ≥ 0.90, Tucker-Lewis Index (TLI) > 0.90, standardized root mean residual (SRMR) < 0.10, and root mean square error approximation (RMSEA) < 0.08. The Bayesian Information Criterion (BIC) was used to compare models, with a smaller BIC value indicating a better fitting model. All models were tested using maximum likelihood estimation. Standard errors and confidence intervals were estimated with 1,000 bias-corrected bootstrapped samples in case of any deviations from normality.

To measure the internal consistency of the WBAL Assessment, we calculated Cronbach’s alpha reliability coefficient for all items and separately for Experiences and Feelings items.

To assess the internal validity of the WBAL Assessment, the correlations among overall WBAL, Experiences and Feelings scores were analyzed. Secondary measures of well-being that could be measured with the WBAL were also examined, including the correlation of the number of frequently positive items (scoring >2 on a scale of 0–4) and mindset positivity (Overall Feelings minus Overall Experiences scores) with overall WBAL, Experiences and Feelings scores.

Correlations of individual items with overall WBAL and each item’s respective factor were examined, as were the correlations among individual items within each Energy level. To test whether items representing different energy levels are capturing unique aspects of well-being beyond those captured by the different factors, we compared correlations across different Energy levels. The strength of correlations between Experiences factors and Feelings factors with different degrees of adjacency in the model were analyzed to test whether the spatial arrangement of factors in the WBAL Lotus representation of the WBAL Model accurately reflects the relationships among related categories of Experiences and Feelings.

To assess the external validity of the WBAL, correlations of overall WBAL scores with PERMA+ and WBA-24 scores were examined, as well as correlations among WBAL factors and corresponding PERMA+ and WBA-24 domains, as measures of convergent validity. To evaluate discriminant validity, the attenuated correlations of WBAL and its factors with PERMA+ and WBA-24 were examined. Attenuated correlations are adjusted for the measures of reliability of the two variables in order to make a ‘true’ estimate of the association between the two constructs. PERMA+ and WBA-24 domains with sufficiently low attenuated correlations to WBAL (95% confidence interval upper bounds not overlapping with 1) and its factors were identified to evaluate discriminant validity between WBAL and the comparator instruments. To evaluate the comparative sensitivity of each tool for assessing well-being spanning from lower to higher well-being, a sub-analysis was performed of the distribution of overall and individual item scores across the study population.

## 3 Results

### 3.1 WBAL model validity: confirmatory factor analysis

To examine the latent variable structure of the model, a confirmatory factor analysis was conducted. Individual items were loaded onto their respective Experiences and Feelings factors, while allowing the residual variances of items of each energy level to correlate with other items of that energy level. The fit of this model was compared to a one-factor model loading all items onto a single wellness factor and to a model that did not allow correlation of residual variances across items’ energy levels.

The model fit statistics are presented for each model in Table 2. The ten-factor and correlated residuals model (referred to as the full model, henceforth) had a lower BIC than the other models and met all criteria for acceptable model fit. Additionally, the ten-factor model without correlated residuals produced a non-positive definite covariance matrix, rendering the solution inadmissible.

**Table 2:** Model fit indices for the three tested models.

**Factor model**	**CFI**	**TLI**	**SRMR**	**RMSEA**	**BIC**
One factor	0.800	0.785	0.069	0.094	2239.173
Ten factors*	0.893	0.870	0.058	0.073	1500.858
Ten factors and correlated residuals	**0.950**	**0.903**	**0.042**	**0.063**	**1168.098**

CFI, comparative fit index; TLI, Tucker-Lewis index; SRMR, standardized root mean residual; RMSEA, root mean squared error approximation; BIC, Bayesian information criterion; *Model was inadmissible.

In the full model, the statistical significance of the parameter estimates (inter-factor correlations, inter-residual correlations, factor loadings) were the key statistics of interest in this analysis: if the 95% bootstrapped confidence interval bounds of the inter-factor correlations did not overlap with zero or one, this was interpreted as the factors being (a) related to each other, and (b) distinct from each other, respectively. Additionally, if the 95% bootstrapped confidence intervals of the regression estimates from each factor onto each item did not overlap with zero, it was implied that the item was significantly loading onto the factor. The correlations between the residual variances were not formally evaluated (although results are presented), because while each correlated residual shares the same energy level (high, mid, or low), each item also has other item-specific error variance of varying degrees, which will affect the strength of these correlations.

**Figure 1:**
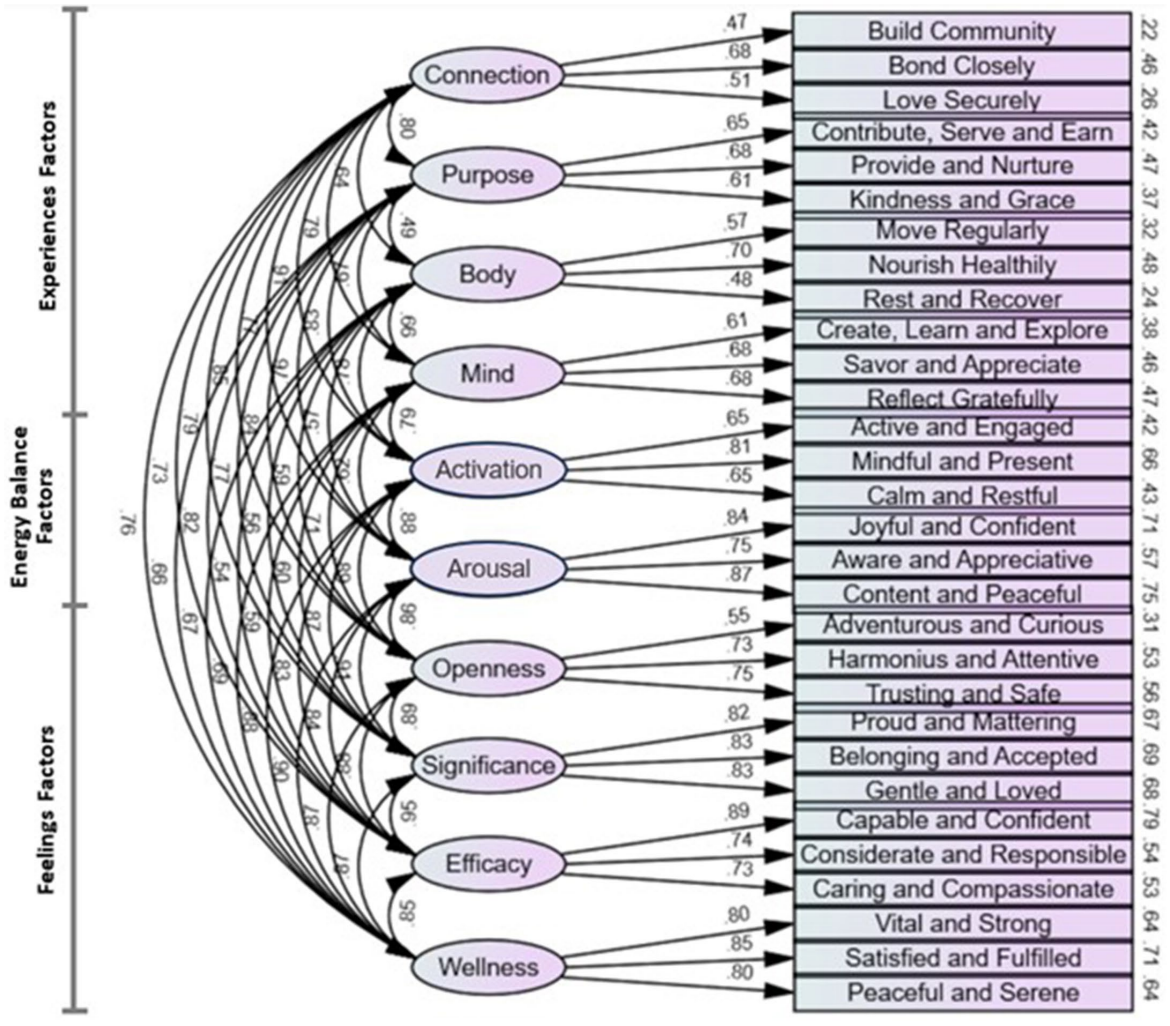
Confirmatory factor analysis results of WBAL scale (residual correlations not shown). Curved double-headed arrows represent correlations. Straight single-headed arrows represent regression loadings. Ovals represent factors. Rectangles represent WBAL survey items. Values on the right of the survey items are squared multiple correlations. All correlations and regression coefficients are significant to *p* < 0.01. Correlations between residual variances with common energy levels are not shown (see Supplementary materials).

The full model, shown without the residual correlations for ease of visualization, is presented in Figure 1, and the full model with residual correlations, as well as all correlations and regression loadings in tabular form, is provided in the Supplementary materials. The confidence interval bounds for all inter-factor correlations in the full model did not overlap with zero or one, indicating that the ten factors are all related to each other, but are distinct from each other and therefore demonstrate internal discriminant validity. Additionally, all of the regression estimates did not have confidence interval bounds overlapping with zero, indicating that all items significantly loaded onto the specified factor. For the residual correlations, the majority of significant correlations were observed between the high-energy items; non-significant residual correlations were retained due to the theoretical justification that residuals should be free to correlate with each other if there is commonality among items within energy levels.

## 4 Internal consistency of the WBAL assessment

To measure the internal consistency of the WBAL Assessment, we calculated Cronbach’s alpha reliability coefficient for all items and separately for Experiences and Feelings items. The overall score included 30 items and had an excellent Cronbach’s alpha of *α* = 0.95. The Feelings and Experiences subscores each contained 15 items. The Feelings score showed excellent internal consistency (*α* = 0.94) while the Experiences score showed good internal consistency (α = 0.87). For comparison, the PERMA+ with 23 items and the WBA-24 with 24 items each had excellent Cronbach’s alpha of *α* = 0.95 in this study.

## 5 Internal validity of the WBAL assessment

Supporting the internal validity of the WBAL Assessment, the overall WBAL score was highly correlated with both the overall Experiences score (*r* = 0.94) and the overall Feelings score (*r* = 0.96). The overall Feelings and overall Experiences scores were also highly correlated (*r* = 0.80). As shown in Table 3, the scores for each factor were highly correlated to the overall WBAL score, consistent with the results from the confirmatory factor analysis. Feelings factors were more highly correlated with overall WBAL score (*r*’s ranging from 0.82 to 0.86) than Experiences factors (*r*’s ranging from 0.61 to 0.75).

**Table 3:** Correlations of WBAL domains and factors with overall WBAL score.

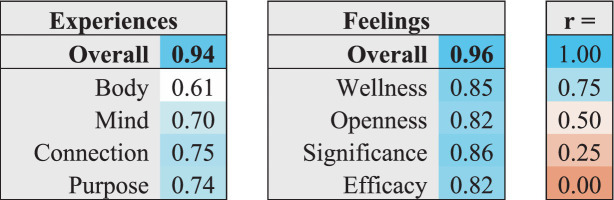

Values are Pearson’s *r*.

We also examined secondary measures of well-being that could be measured with the WBAL Assessment. The number of frequently positive items (scoring >2 on a scale of 0 to 4) strongly correlated with overall WBAL score (*r* = 0.96), which suggests that this could be a meaningful summary score. Mindset positivity (Overall Feelings minus Overall Experiences scores) correlated with Overall Feelings (*r* = 0.58) but did not correlate with Overall Experiences (*r* = −0.03) and showed a weak correlation to Overall WBAL score (*r* = 0.33).

### 5.1 Energy levels

The relationship within energy levels in the model, as described in Table 4, showed strong correlations between Experiences and Feelings of the same energy level, consistent with the results from the confirmatory factor analysis. Each Experiences Activation energy level correlated strongly with the corresponding Feelings Arousal energy level: Active/Engaged Experiences and Joyful/Confident Feelings were strongly correlated (Active energy levels, *r* = 0.66), Mindful/Present Experiences and Aware/Appreciative Feelings were highly correlated (Mindful energy level, *r* = 0.73), and Calm/Restful Experiences were highly correlated with Content/Peaceful Feelings (Calm energy level, *r* = 0.73).

**Table 4:** Correlations between Experiences Activation Energy Levels and the corresponding Feelings Arousal Energy Level.

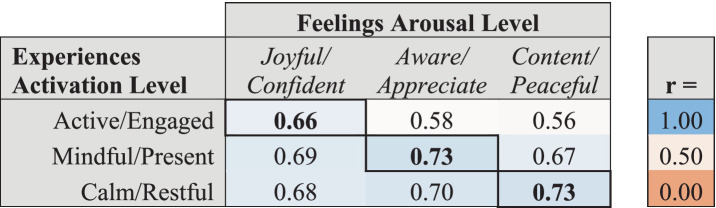

Bold values represent corresponding energy levels of WBAL Experiences and Feelings. Values are Pearson’s *r*.

As shown in Table 5, each assessment item was significantly correlated with other items of the same energy level within the corresponding Experiences and Feelings domain. Feelings items of a given Arousal Energy level were generally more strongly correlated with all Feelings items of the same Arousal Energy level (*r*’s ranging from 0.67 to 0.82) than individual Experiences items were with all Experiences items of the same Activation Energy level (*r*’s ranging from 0.54 to 0.72).

**Table 5:** Correlations of energy level assessment items within each factor with the overall mean scores for the corresponding energy level across all items within the corresponding Experiences and Feelings domains.

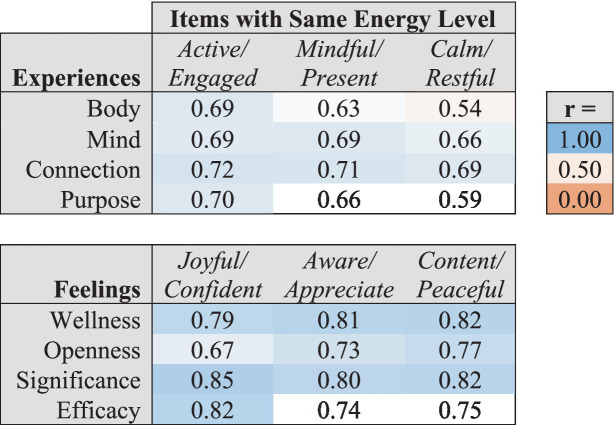

Values are Pearson’s *r*.

As shown in Table 6, each item within each factor representing the energy level of that factor (i.e., Activation levels of Experiences and Arousal levels of Feelings) was highly correlated with its factor (*r*’s between 0.70 and 0.89). Correlations between items of different energy levels within each factor were lower than with the factor overall (*r*’s between 0.20 and 0.71), with the lowest correlations observed between Active and Calm energy levels within each factor.

**Table 6:** Correlations of energy level assessment items with the overall scores for each factor to which they are mapped, and between items of different energy levels within factors.

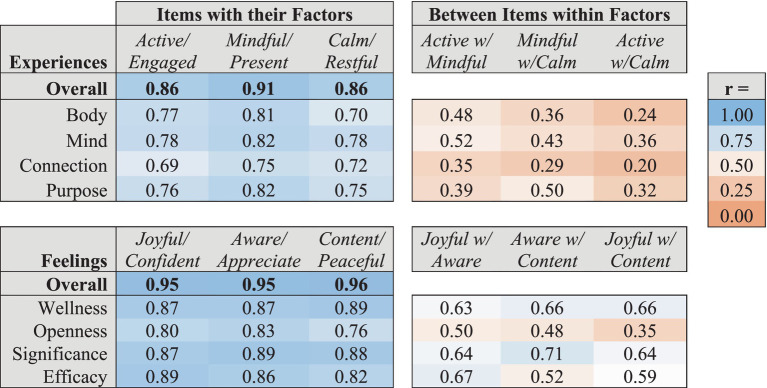

Values are Pearson’s *r*.

For example, Active and Calm Experiences Activation levels were very weakly correlated (*r*’s ranging from 0.20 to 0.36). Correlations between Mindful and Calm Experiences Activation levels were low (*r*’s ranging from 0.29 to 0.50). And correlations between Active and Mindful Experiences Activation levels were also modest (*r*’s ranging from 0.35 to 0.52). Correlations across Feelings Arousal energy levels were somewhat higher (*r*’s ranging from 0.35 to 0.67), but still consistently lower than their correlations with their factor overall. This is consistent with the confirmatory factor analysis, showing that the different energy levels are capturing unique aspects of well-being within each factor beyond those captured by the different factors.

### 5.2 Factor adjacency

The strength of correlations between Experiences factors and Feelings factors with different degrees of adjacency in the model were analyzed to test whether the spatial arrangement of factors in the WBAL Lotus graphical framework accurately represents the relationships between different categories of Experiences and Feelings. As shown in Table 7, each Experiences factor was somewhat more strongly correlated with its two most closely adjacent Feelings factors than with their two more distant Feelings factors. As predicted by the WBAL Model, this indicates somewhat closer associations between Experiences factors and Feelings factors that are positioned more closely adjacent in the WBAL Lotus framework.

**Table 7:** Correlations between adjacent versus distant Experiences and Feelings factors.

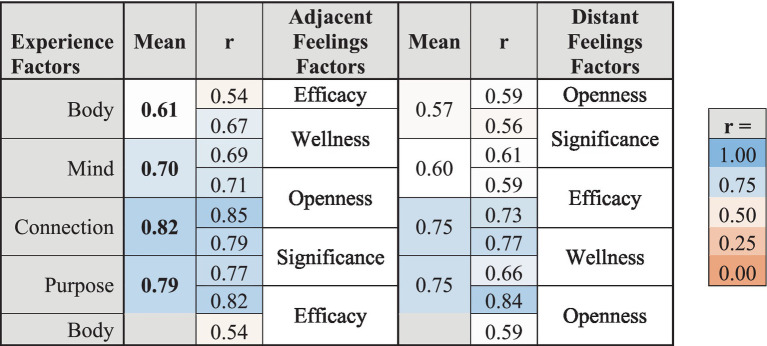

Values are Pearson’s *r*.

## 6 External validity of the WBAL assessment

The second aim of the study was to evaluate convergent validity of the WBAL Model and Assessment with the constructs of well-being captured by already validated tests of well-being, PERMA+ and WBA-24, as well as discriminant validity between WBAL and these comparator assessments.

### 6.1 Convergent validity

As shown in Table 8, overall WBAL scores correlated closely with PERMA+ (*r* = 0.80) and WBA-24 (*r* = 0.75). The PERMA+ and WBA-24 were most strongly correlated with WBAL Feelings score (*r* = 0.83 and 0.79 respectively) and not as strongly correlated with WBAL Experiences (*r* = 0.66 and 0.62 respectively). The WBAL summary measure of number of frequently positive items (scoring >2 on a scale of 0 to 4) also correlated highly with overall PERMA+ (*r* = 0.72) and WBA-24 (*r* = 0.68).

**Table 8:** Correlations of WBAL Overall, Experiences and Feelings with previously validated comparator constructs.

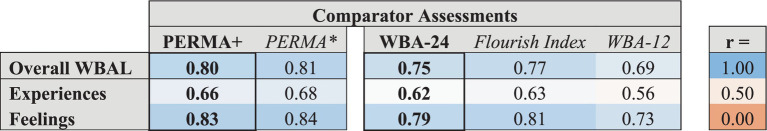

Values are Pearson’s *r*. * PERMA excluding PERMA+ items for health, negative emotion and loneliness.

The WBA-24 was developed based on two previously existing scales, so we also investigated and found strong correlations between overall WBAL score and overall scores for the Flourishing Index (*r* = 0.77) and Well-Being Assessment 12-items (*r* = 0.69). The PERMA+ is an expansion of the original PERMA scale, which also showed a strong correlation to the WBAL overall score (*r* = 0.81).

Because the PERMA+ and WBA-24 constructs each assess related aspects of well-being, we calculated the correlations of each WBAL factor with overall PERMA+ and WBA-24 scores, summarized in Table 9. Correlations of PERMA+ and WBA-24 with WBAL Experiences factors were consistently weaker than with WBAL Feelings factors. Both PERMA+ and WBA-24 were most strongly correlated with WBAL Feelings of Significance and Wellness.

**Table 9:** Correlations between WBAL factors and PERMA+ and WBA-24 domains.

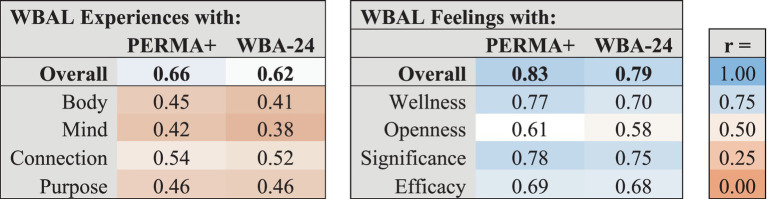

Values are Pearson’s *r*.

As shown in Table 10, other notably strong correlations between WBAL factors and PERMA+ domains include overall positive WBAL Feelings with PERMA+ Positive Emotion (*r* = 0.82), Meaning (*r* = 0.79) and Happiness (*r* = 0.77). The strongest correlations between WBAL factors and WBA-24 domains were overall positive WBAL Feelings with WBA-24 Life Satisfaction & Evaluation (*r* = 0.73), Meaning & Purpose (*r* = 0.74) and Affect (*r* = 0.71). In a sub-analysis of raw correlations among WBAL factors and PERMA+ and WBA-24 domains, the strongest correlations were WBAL Feelings of Significance with PERMA+ Meaning (*r* = 0.76) and Relationships (*r* = 0.69) and with WBA-24 Meaning & Purpose (*r* = 0.72) and Relationships (*r* = 0.65). Comparator Domains with the weakest observed correlations with WBAL were PERMA+ Negative Emotion and Loneliness (*r*’s = 0.40), and WBA-24 Financial Evaluation and Stability (*r* = 0.30), each representing concepts not assessed by WBAL.

**Table 10:** Correlations between WBAL domains and PERMA+ and WBA-24 domains.

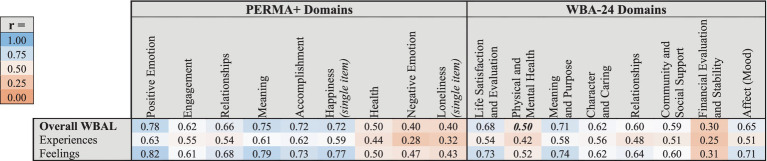

Values are Pearson’s *r*.

### 6.2 Discriminant validity

Discriminant validity was first tested by correlating each WBAL factor with each domain from the PERMA+ and WBA-24 questionnaires. For each WBAL factor, if the upper bounds of the 95% confidence intervals surrounding the correlation coefficient did not overlap with 1 for any PERMA+ or WBA-24 domain, then the factor was considered to show discriminant validity. Prior to attenuating correlations, no factor had correlation confidence interval bounds crossing 1, so all WBAL factors were implied to show discriminant validity.

To fully account for potential measurement error and imperfect reliability, discriminant validity was then tested by calculating attenuated correlations between each WBAL factor and each domain from the PERMA+ and WBA-24 questionnaires. For each WBAL factor, if the upper bounds of the 95% confidence intervals surrounding the attenuated correlation coefficient did not overlap with 1 for any PERMA+ or WBA-24 domain, then the factor was considered to show discriminant validity. Each WBAL factor overlapped with an average of 2.7 out of 17 PERMA+ and WBA-24 domains (minimum = 0, maximum = 6), implying that there is large degree of, but not complete, discriminant validity. Table 11 shows the attenuated correlations between WBAL factors and PERMA+ and WBA-24 domains.

**Table 11:** Attenuated correlations between WBAL factors and PERMA+ and WBA-24 domains.

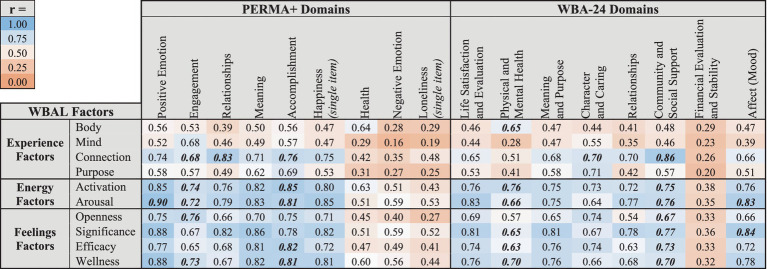

Non-discriminate correlations are bold italicized. Values are Pearson’s *r*.

The PERMA+ domains most discriminant from WBAL factors were Negative Emotion and Loneliness, and the WBA-24 domain most discriminant from WBAL factors was Financial Stability and Evaluation.

WBAL Experiences factors showed discriminant validity across nearly all PERMA+ and WBA-24 domains (63 of 68 correlations). All WBAL Experiences factors showed discriminant validity with all PERMA+ domains except WBAL Connection with PERMA+ Engagement, Relationships and Accomplishments. All WBAL Experiences factors showed discriminant validity with all WBA-24 domains except WBAL Body with WBA-24 Health and Physical Function, and WBAL Connection with WBA-24 Community and Social Support.

WBAL Feelings factors were somewhat less likely to show discriminant validity across PERMA+ and WBA-24 domains (56 of 68 comparisons). No WBAL Feelings factors showed discriminant validity with WBA-24 Community and Social Support and, among WBAL Feelings factors, only Openness showed discriminant validity with WBA-24 Health and Physical Function. WBAL Efficacy and Wellness did not show discriminant validity with PERMA+ Accomplishment, and WBAL Openness and Wellness did not show discriminant validity with PERMA+ Engagement.

WBAL energy range factors were least likely to show discriminant validity with PERMA+ and WBA-24 domains (24 of 34 comparisons). Neither WBAL Experiences Activation energy range nor Feelings Arousal energy range showed discriminant validity with PERMA+ Engagement or Accomplishment, or WBA-24 Health and Physical Function or Community and Social Support.

### 6.3 Instrument sensitivity

**Figure 2:**
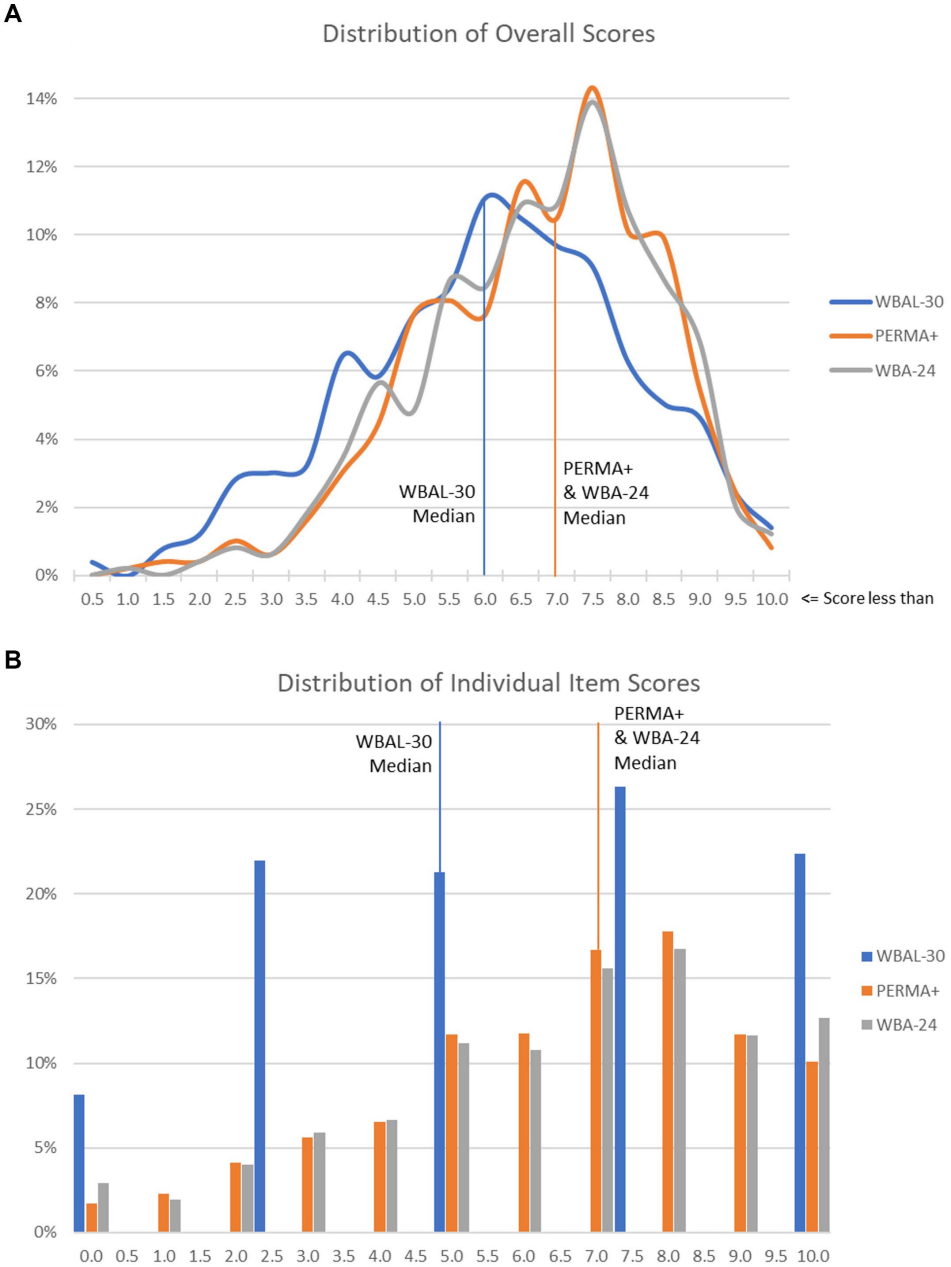
**(A)** Distribution of WBAL versus PERMA+ and WBA-24 overall scores (WBAL normalized to a 0–10 scale). **(B)** Distribution of WBAL versus PERMA+ and WBA-24 individual item scores (WBAL normalized to a 0–10 scale).

To evaluate the relative sensitivity of each tool for assessing well-being across the spectrum from lower to higher well-being, a sub-analysis was performed of the distribution of overall and individual item scores across the study population (with WBAL normalized to a 0–10 scale for comparison with the comparators’ 11-point Likert scales), as shown in Figure 2. The distribution of PERMA+ and WBA-24 scores were very similar and each skewed higher than the distribution of WBAL scores, with median scores approximately a point higher than WBAL’s.

## 7 Discussion

This study confirms the validity of the WBAL Model of Well-Being Balance and Lived Experiences, and the internal consistency and validity of the WBAL Assessment. Compared to two well-validated “gold standard” integrative assessments of human flourishing, PERMA+ and WBA-24, the WBAL Assessment demonstrated good external validity, including high convergent validity overall as well as high discriminant validity among factors and domains.

### 7.1 WBAL model validity

The factor model with the best fit meeting all validity criteria loaded individual items onto their respective Experiences and Feelings factors, while allowing residual variances of each energy level to correlate with other items of the same energy level, but not different energy levels. This confirms the validity of the overall WBAL Model construct, including that: (1) the individual Experiences factors (Body, Mind, Connection, Purpose) and Feelings factors (Openness, Significance, Efficacy, Wellness), as well as the Experiences Activation Range and Feelings Arousal Range factors, are all related to each other but distinct from each other; (2) the individual items load significantly onto their respective factors; and (3) items of similar Activation or Arousal energy levels correlate to varying degrees of significance.

### 7.2 Internal consistency and validity

The WBAL Assessment was demonstrated to be internally consistent, with a high Crohnbach’s alpha equal to those observed for each of the two comparator instruments. The WBAL Assessment demonstrated high internal validity, with high correlations among the overall WBAL score, and overall positive Feelings and Experiences scores. High correlations were seen with overall WBAL scores for each Experiences and Feelings factor, with Feelings factors being more strongly correlated.

The energy levels within each factor, including Experiences Activation levels and Feelings Arousal levels, were each highly correlated with their factor and with other items of the same energy level. In contrast, correlations between differing energy levels within each factor were substantially lower. This suggests that items of different energy levels within factors are measuring distinct contributors to well-being, and that including items to assess a balance of high, moderate and low Activation and Arousal levels extends the model’s utility.

### 7.3 Convergent and discriminant validity

The WBAL Assessment demonstrated high convergent validity in comparison to the PERMA+ and WBA-24 well-being instruments (*r* = 0.80 and 0.75 respectively), indicating that the WBAL is a valid alternative integrative tool for assessing human flourishing that measures a similar overall concept of well-being. The correlation of the WBAL Feelings scores with each of these comparators (*r* = 0.83 and 0.79 respectively) was higher than for WBAL Experiences scores (*r* = 0.66 and 0.68 respectively), suggesting that the WBAL Assessment’s measurement of positive experiences that contribute to overall well-being is a key difference from the comparator well-being measures, whereas positive Feelings as measured by WBAL is a more direct measure of subjective feelings of flourishing. This result can be expected due to the comparator measures being focused on subjective feelings of well-being, without relating these directly to an individual’s recent experiences.

In particular, overall positive Feelings as measured by the WBAL Assessment correlated closely with comparator domains related to Life Satisfaction, Happiness, and Positive Emotions/Affect, suggesting that WBAL Feelings summary score is a valid independent measure of overall subjective well-being as conceptualized by the hedonic tradition. Unlike the comparator assessments, the WBAL evaluates 12 distinct categories of Feelings of well-being. Having a more complete view of the specific Feelings underlying an individual’s sense of Life Satisfaction and Happiness may aid in developing an intervention plan to improve the individual’s subjective well-being.

The correlations between components of the WBAL Assessment with comparator assessments were lower than correlations between overall well-being as measured by each tool. This suggests that while the WBAL is measuring the same overall concept of well-being as PERMA+ and WBA-24, it is measuring a different conceptualization of well-being. Notably, PERMA+ and WBA-24 do not directly assess positive experiences, and include measures of negative affect and emotions, such as loneliness, while WBA also includes measures of financial stability. In contrast, WBAL measures both Experiences and Feelings, only measures positive contributors to well-being and does not evaluate financial stability directly. Accordingly, WBAL Experience factors were most likely to be discriminant from comparator domains, and the comparator domains most discriminant from WBAL factors were PERMA+ Negative Emotion and Loneliness and WBA-24 Financial Evaluation and Stability.

Certain domains do, however, appear to be measuring very similar aspects of well-being across these instruments, despite using a different methodology with different prompts. For example, the attenuated correlations of multiple WBAL Experience factors were non-discriminant from closely corresponding comparator domains, including: WBAL Connection with PERMA+ Engagement, Relationships and Accomplishments; WBAL Body with WBA-24 Health and Physical Function; and WBAL Connection with WBA-24 Community and Social Support.

The differing distributions of overall and individual item scores are another key difference among instruments, with the comparator assessments skewing higher, and further from center, than the WBAL, suggesting that the WBAL may be more sensitive to discriminate among higher levels of well-being than comparator instruments. Arithmetically, this may result from inclusion in the comparator instruments of emotions with negative valence, which are inversely correlated with positive emotions despite a degree of independence (Diener and Emmons, 1984), and thereby increase the quantitative weight of these detractors from well-being in the overall scores.

## 8 Practical implications

This study confirms that the WBAL Model is a valid construct and the WBAL Assessment is a valid instrument for evaluating well-being and human flourishing. The WBAL Model and Assessment generally performed as postulated, confirming key similarities and differences from comparators. Some WBAL factors closely correspond with comparator domains, which have previously been well-demonstrated to be important contributors to subjective well-being and flourishing. Key differences of WBAL from PERMA+ and WBA-24 include the evaluation of positive lived experiences alongside positive feelings, and assessment of a balance of low to high arousal and activation levels across a full spectrum of positive feelings and experiences.

Having a valid tool to assess the frequency and range of positive experiences in an individual’s life and relate these to the frequency and range of their positive feelings enables the identification of specific modifiable contributors to well-being which can form the basis of a personalized well-being intervention plan. By focusing on a comprehensive set of discrete categories of positive experiences that have been demonstrated to enhance well-being, this new model can enable individuals to identify and pursue specific experiences with the greatest promise to improve their well-being.

The WBAL Assessment may also be useful to more precisely identify gaps in positive well-being for individuals who report low life satisfaction, subjective well-being or positive affect, as measured by abbreviated measures such as the Satisfaction With Life Scale (Diener et al., 1985), Subjective Happiness Scale (Lyubomirsky and Lepper, 1999), or Positive and Negative Affect Scale (PANAS) (Watson et al., 1988). Because the WBAL Assessment covers such a broad spectrum of categories of positive experiences and feelings without precision within each category, the design of personalized interventions may benefit from further assessment interrogating more specific aspects of well-being indicated by WBAL to be of interest, or using localized or contextual models with greater relevance to the individual’s particular situation and context (Alexandrova, 2017; Mitchell and Alexandrova, 2021).

By evaluating well-being across a range of activation and arousal levels, the WBAL Assessment supports a more balanced approach to the pursuit of well-being across the full range of well-being contributors. Taken together, the WBAL Model and Assessment support design of more personalized intervention programs with greater potential to enhance positive well-being.

The strong correlation of total number of frequently felt or experienced items with overall WBAL score, as well as each of the comparator assessments, suggests that this could be a meaningful independent summary score of well-being. In turn, focusing interventions on raising the frequency of positive Experiences or Feelings not frequently experienced (i.e., with scores <2) may be an effective way to improve overall positive well-being. Prior research has indicated that cultivating positive emotions can increase resilience in response to negative emotional experiences (Tugade and Fredrickson, 2004). The number of frequently felt and experienced WBAL categories may be a useful metric to investigate whether a broader range of positive Experiences and Feelings corresponds with higher resilience in response to negative life events, and conversely, whether well-being that is narrowly focused on fewer positive contributors to well-being is more fragile in the face of negative life events.

Furthermore, the extent to which overall Feelings scores exceed overall Experiences scores appears to be a meaningful measure of Mindset positivity, which correlates strongly with overall positive Feelings and not with positive Experiences. This finding suggests that a more positive Mindset, independent of the breadth and frequency of positive Experiences, is associated with more positive Feelings about those experiences, which in turn corresponds with higher overall well-being. Mindset positivity may also be important for an individual’s response to stressful life events, and thereby mitigate the well-being impact of these events. Positive mindset around stressful experiences has been shown to result in a smaller detriment to well-being, with a decoupling between experiences and feelings (Crum et al., 2013; Park et al., 2018; Young et al., 2021). The WBAL Mindset positivity metric may be a useful independent measure of the efficacy of interventions targeting positive mindset (i.e., mindfulness, presence, gratitude, forgiveness, intentions, etc.) as a means to improve overall subjective well-being and increase well-being resilience (Khoury et al., 2015; Davis et al., 2016).

## 9 Limitations and future directions

Due to an inability to recontact de-identified respondents, this study did not evaluate test–retest reliability of the assessment, which should be the subject of future research.

The WBAL Assessment provides a more granular view of the relationships among individuals’ Experiences and Feelings, as a tool to understand specific gaps in individuals’ well-being that can guide personalized interventions. However, the current study only demonstrates correlations among positive Experiences and their related positive Feelings, not causality between factors. Prior research indicates that causality is likely to be bidirectional, i.e., engaging in more positive experiences increases positive feelings of well-being (Buecker et al., 2021), and positive affect leads to more healthy behaviors (Pressman et al., 2019), well-being and success behaviors (Lyubomirsky et al., 2005a). There is strong evidence that improving mindfulness, and engaging in mindful activities, can increase well-being (Fredrickson et al., 2008; Walsh et al., 2019).

Furthermore, evidence supports that health and well-being can be improved by engaging in specific positive activities, such as sleep (Scott et al., 2021), social connection (Martino et al., 2015) or acts of kindness (Lyubomirsky et al., 2005b; Curry et al., 2018). Future studies are needed to confirm causalities among positive experiences and positive feelings of well-being and to evaluate the WBAL Assessment as a programmatic tool to identify and prioritize specific types of positive experiences that are most likely to improve positive well-being and flourishing for an individual or population.

Because the WBAL Assessment only directly evaluates positive Experiences and positive Feelings, the instrument only indirectly measures how negative Experiences and Feelings affect overall well-being. Future studies are needed to evaluate the effect of negative experiences and life stressors on the validity and interpretation of the WBAL Assessment, especially with regard to negative feelings.

The WBAL Assessment does not include direct measures of work engagement or productivity, nor financial security and stability, and measures of work engagement or job satisfaction were not included as comparators in this study. Overall WBAL score was strongly correlated with PERMA+ Accomplishment (*r* = 0.72) but not WBA-24 Financial Evaluation and Stability (*r* = 0.30), suggesting a complex relationship of positive well-being with work engagement and financial security, as seen in prior research on financial well-being (Collins and Urban, 2021). Further study is needed to understand the relationship of overall well-being as measured by the WBAL with work engagement, job satisfaction and financial security.

For ease of administration, each item of the 30-item Well-Being Balance and Lived Experiences Assessment (WBAL-30) studied here integrates multiple discrete subitems into each individual assessment item. Further study is warranted to determine the validity and utility of a more comprehensive 90-item version of the WBAL Assessment (WBAL-90) that has been developed in accordance with the WBAL Model to evaluate each category of positive Experiences and Feelings that contribute to well-being with even more specificity. The aim of this more comprehensive WBAL-90 is to enable a practitioner to understand the underlying discrete contributors to overall well-being even more granularly to support a more robust dialogue with individuals about opportunities to improve their well-being and to design more tailored and personalized interventions for individuals.

Finally, as with other similar well-being assessment tools, WBAL implicitly assumes normative values regarding the meaning of well-being. Caution is therefore warranted to avoid normative judgment when interpreting responses of individuals whose norms and values may differ. The tool does not assign relative significance to any of the aspects of positive well-being measured, so interpretation must allow for respondents to assign different degrees of importance to different aspects of their well-being, based on their personal value judgments.

## Data availability statement

The raw data supporting the conclusions of this article will be made available by the authors, without undue reservation.

## Ethics statement

The studies involving humans were approved by Solutions IRB Institutional Review Board. The studies were conducted in accordance with the local legislation and institutional requirements. The ethics committee/institutional review board waived the requirement of written informed consent for participation from the participants or the participants’ legal guardians/next of kin because the study was determined to present no or minimal risk to research subjects.

## Author contributions

AM: Conceptualization, Methodology, Project administration, Validation, Writing – original draft, Writing – review & editing. CB: Formal analysis, Methodology, Software, Validation, Visualization, Writing – original draft, Writing – review & editing. TN: Conceptualization, Data curation, Formal analysis, Funding acquisition, Investigation, Methodology, Project administration, Resources, Supervision, Validation, Visualization, Writing – original draft, Writing – review & editing.

## Figures and Tables

**Appendix FIGURE 1:**
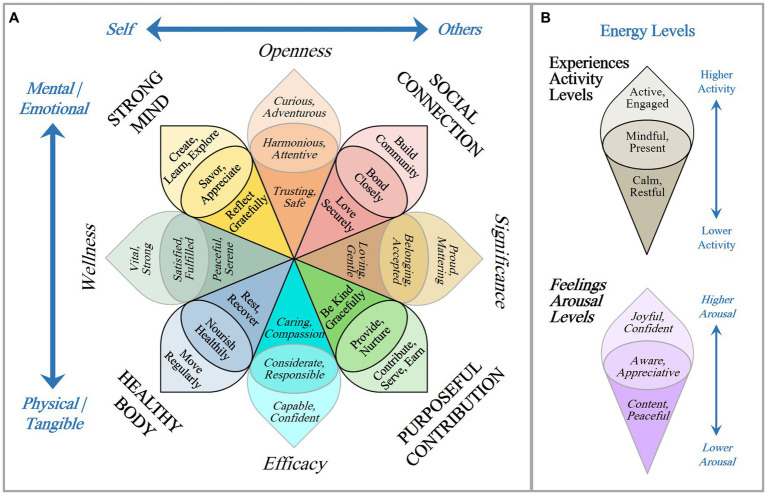
**(A)** The WBAL Model. Factors within the Experiences domain are denoted with all capitalized letters and factors within the Feelings domain are denoted with only the first letter capitalized. **(B)** Activation and Arousal Energy Levels within WBAL Experiences and Feelings domains, respectively.

## Appendix A: well-being balance and lived experiences (WBAL) model

Drawing together findings across positive psychology and well-being fields of research, the WBAL Model posits that our subjective sense of well-being arises from positive life experiences including caring for ourselves mentally and physically by attending to our minds and bodies, and engaging with others emotionally and tangibly by nurturing positive social relationships and engaging in purposeful activities that contribute to others’ well-being. Additionally, the WBAL Model aims to encompass the full range of hedonic and eudaimonic positive experiences and feelings, which balances mental and physical activity and stimulation with savoring and mindful engagement, and with rest and reflection. Each item of positive experiences corresponds with a body of evidence supporting the positive impact of these activities and experiences on subjective well-being and human flourishing.

The WBAL Model, based on individuals’ recent frequency of a range of positive experiences and positive feelings, further postulates that more frequent positive experiences overall should correspond with more frequent positive feelings overall, and that together, these should correspond closely with an individual’s self-reported subjective well-being or flourishing. Additionally, the model speculates that specific types of positive experiences are more likely to correspond with specific types of positive feelings, and that the more categories of positive experiences and feelings an individual frequently has, the greater their overall well-being will be, in an upward spiral of positivity in accordance with the broaden and build theory of positive emotions (Fredrickson, 2001).

The WBAL Model is illustrated in Appendix Figure 1a as a lotus flower (WBAL Lotus) that interweaves items of positive experiences with items of related positive feelings. The model has two (2) domains of Experiences and Feelings, with eight (8) factors including four (4) Experiences factors (Body, Mind, Connection, and Purpose) and four (4) Feelings factors (Wellness, Openness, Significance, and Efficacy), represented as separate petals of the lotus flower. WBAL scores represent an individual’s recall upon reflection of recent positive experiences and feelings in their lives.

Within each factor, energy levels denote relative Activation levels of Experiences and Arousal levels of Feelings, with lower activation and arousal levels at the center and higher activation and arousal levels along the outside of the petals of the WBAL Lotus. As shown in Appendix Figure 1b, for Experiences these energy levels are referred to as Activation levels: Active/Engaged, Mindful/Present, and Calm/Restful. For Feelings these Energy levels are referred to as Arousal levels: Joyful/Confident, Aware/Appreciative, and Content/Peaceful. Each discrete energy level within a factor is a distinct contributor to well-being that corresponds to an item on the WBAL Assessment. Additionally, the WBAL Assessment includes three items of each energy level to assess the overall range of Experience Activation levels and three corresponding items to assess the overall range of Feelings Arousal levels.
