# Supplementary material for: Well-being balance and lived experiences assessment: a valid, comprehensive measure of positive well-being
Source: Front Psychol. 2024 Aug 8;15:1396543. doi: 10.3389/fpsyg.2024.1396543 (PMC11339688; doi:10.3389/fpsyg.2024.1396543)
Supplement: Supplementary file 1 [file Table_1.DOCX]

Supplemental Figure 1. Confirmatory factor analysis results of WBAL Scale. Curved double-headed arrows represent correlations. Straight single-headed arrows represent regression loadings. Ovals represent factors. Rectangles represent WBAL survey items. Values on the right of the survey items are squared multiple correlations. All correlations and regression coefficients are significant to p < .01.


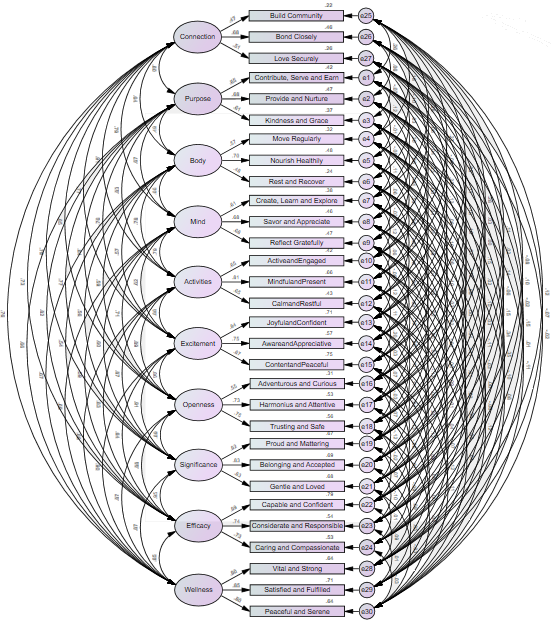


Given the difficulty to read the values of all the parameter estimates in Supplemental Figure 1, correlations between factors are presented in Supplemental Table 1, and correlations between residual variances for each energy level are presented in Supplemental Tables 2-4.

Supplemental Table 1. Confirmatory Factor Analysis Inter-Factor Correlations

| Factor | 1 | 2 | 3 | 4 | 5 | 6 | 7 | 8 | 9 | 10 |
| --- | --- | --- | --- | --- | --- | --- | --- | --- | --- | --- |
| 1. Excitement | - |  |  |  |  |  |  |  |  |  |
| 2. Activities | .879 | - |  |  |  |  |  |  |  |  |
| 3. Wellness | .897 | .879 | - |  |  |  |  |  |  |  |
| 4. Connection | .769 | .911 | .765 | - |  |  |  |  |  |  |
| 5. Efficacy | .842 | .835 | .847 | .733 | - |  |  |  |  |  |
| 6. Significance | .909 | .869 | .871 | .792 | .947 | - |  |  |  |  |
| 7. Openness | .857 | .890 | .866 | .854 | .885 | .891 | - |  |  |  |
| 8. Mind | .621 | .785 | .688 | .787 | .586 | .605 | .713 | - |  |  |
| 9. Body | .568 | .778 | .672 | .641 | .543 | .557 | .587 | .658 | - |  |
| 10. Purpose | .757 | .833 | .660 | .804 | .820 | .768 | .835 | .668 | .493 | - |

*Note.* All correlations are significant to *p* < .01.

Supplemental Table 2. Correlations between Residual Variances of High Energy Items.

| Item | 1 | 2 | 3 | 4 | 5 | 6 | 7 | 8 | 9 | 10 |
| --- | --- | --- | --- | --- | --- | --- | --- | --- | --- | --- |
| 1. Active and Engaged | - |  |  |  |  |  |  |  |  |  |
| 2. Joyful and Content | .116* | - |  |  |  |  |  |  |  |  |
| 3. Contribute, Serve, and Earn | .226** | .116 | - |  |  |  |  |  |  |  |
| 4. Move Regularly | .529** | .241** | .117* | - |  |  |  |  |  |  |
| 5. Create, Learn, and Explore | .120 | .096 | .198** | .106 | - |  |  |  |  |  |
| 6. Build Community | .268** | .052 | .256** | .180** | .278** | - |  |  |  |  |
| 7. Adventurous and Curious | .124* | .028 | .103 | .077 | .177** | .277** | - |  |  |  |
| 8. Proud and Mattering | .034 | .111 | .145* | .087 | .018 | .109* | .068 | - |  |  |
| 9. Capable and Confident | .089 | -.004 | -.060 | .033 | -.108 | -.076 | -.061 | .067 | - |  |
| 10. Vital and Strong | .381** | .066 | .152* | .353** | .062 | .122* | .076 | .087 | .090 | - |

*Note.* * = *p* < .05, ** = *p* < .01. P values are calculated using bias-corrected bootstrapped standard errors and confidence intervals.

Supplemental Table 3. Correlations between Residual Variances of Mid Energy Items.

| Item | 1 | 2 | 3 | 4 | 5 | 6 | 7 | 8 | 9 | 10 |
| --- | --- | --- | --- | --- | --- | --- | --- | --- | --- | --- |
| 1. Mindful and Present | - |  |  |  |  |  |  |  |  |  |
| 2. Aware and Appreciative | .114 | - |  |  |  |  |  |  |  |  |
| 3. Provide and Nurture | -.005 | .058 | - |  |  |  |  |  |  |  |
| 4. Nourish Healthily | .063 | .087 | -.005 | - |  |  |  |  |  |  |
| 5. Savor and Appreciate | .061 | .036 | .058 | .057 | - |  |  |  |  |  |
| 6. Bond Closely | .052 | .112 | .062 | -.096 | .059 | - |  |  |  |  |
| 7. Harmonious and Attentive | .047 | .069 | .009 | .015 | .521** | .030 | - |  |  |  |
| 8. Belonging and Accepted | -.115 | .038 | .024 | .004 | -.039 | .016 | -.123* | - |  |  |
| 9. Considerate and Responsible | -.012 | .118 | .155* | -.109 | .014 | .102 | .013 | -.099 | - |  |
| 10. Satisfied and Fulfilled | .057 | .118 | .009 | -.101 | -.064 | -.069 | .003 | .050 | .008 | - |

*Note.* * = *p* < .05, ** = *p* < .01. P values are calculated using bias-corrected bootstrapped standard errors and confidence intervals.

Supplemental Table 4. Correlations between Residual Variances of Low Energy Items.

| Item | 1 | 2 | 3 | 4 | 5 | 6 | 7 | 8 | 9 | 10 |
| --- | --- | --- | --- | --- | --- | --- | --- | --- | --- | --- |
| 1. Calm and Restful | - |  |  |  |  |  |  |  |  |  |
| 2. Content and Peaceful | .204** | - |  |  |  |  |  |  |  |  |
| 3. Kindness and Grace | .037 | -.101 | - |  |  |  |  |  |  |  |
| 4. Rest and Recover | -.032 | .014 | -.077 | - |  |  |  |  |  |  |
| 5. Reflect Gratefully | -.091 | .044 | -.041 | -.024 | - |  |  |  |  |  |
| 6. Love Securely | .222** | .099 | -.028 | .028 | -.040 | - |  |  |  |  |
| 7. Trusting and Safe | .108 | -.029 | -.098 | .030 | -.051 | -.001 | - |  |  |  |
| 8. Gentle and Loved | .079 | -.133 | .148* | -.040 | .002 | -.021 | -.021 | - |  |  |
| 9. Caring and Compassionate | -.041 | -.087 | .344** | -.033 | .033 | -.017 | -.095 | .013 | - |  |
| 10. Peaceful and Serene | .036 | .111 | -.112* | .122 | .122 | -.020 | .122 | -.028 | -.028 | - |

*Note.* * = *p* < .05, ** = *p* < .01. P values are calculated using bias-corrected bootstrapped standard errors and confidence intervals.
